# Detection of electron beam energy variations using a computed radiography system

**DOI:** 10.1120/jacmp.v10i4.2911

**Published:** 2009-10-15

**Authors:** Yang C. Cai, Yuanyuan Ge, Damian Bernard, Julius Turian, James C. H. Chu

**Affiliations:** ^1^ Department of Medical Physics Rush University Medical Center Chicago Illinois 60612

**Keywords:** electron energy consistency, computed radiography, storage phosphor

## Abstract

A method to evaluate the electron beam energy constancy by employing the computed radiography (CR) system has been developed. In this method, a right triangular plastic wedge is used to produce a curve of the CR storage phosphor plate signal versus the wedge thickness. The curve, which resembles the percentage depth ionization curve of the clinical electron beams, can be used to derive the energy constancy metric ${\text{EC}}_{50}$. The sensitivity of the method was tested using polystyrene sheets of variable thicknesses. For electron energies up to 12 MeV, energy changes induced by 1.5 mm thick polystyrene can be detected, while a 2.3 mm thick polystyrene sheet is required for higher energies. The measurements were carried out over a two‐year period. The results showed a good reproducibility with the use of the same CR plate and cassette, and without the requirement of calibration procedures. The two‐year range of the ${\text{EC}}_{50}$ was within the 99% confidence intervals, and the standard deviation of the ${\text{EC}}_{50}$ was measured to be from 0.3 to 0.4 mm for different beam energies. This technique provides an efficient and accurate method to perform the electron beam energy check and could be used by centers equipped with the CR system without requiring additional detection devices.

PACS number: 87.56.Fc

## I. INTRODUCTION

For medical accelerators, AAPM TG40^(1)^ recommends that the electron beam quality be checked monthly to ensure consistency with commissioning data. The tolerance is ±2.0mm shift of the therapeutic depth in the electron percent depth dose curve. In order to avoid the tediousness of measuring the actual PDD curve in water or plastic phantom and to make this monthly energy constancy check practical, several techniques have been developed. Some of these use wedge attenuators. The techniques that use wedge attenuators fall into two categories.

- Techniques based on the linear dependency between the beam energy and the electron range
  A large scale ionization chamber, either cylindrical or parallel plate, is irradiated under a wedge attenuator.^(^ ^2^ ^,^ ^3^ ^)^ Electrons of a specific energy can only penetrate through the part of the wedge that has a thickness smaller than the electron range. The total collected ionization signal is then proportional to the electron energy.
- Techniques whereby a curve is obtained by placing a wedge attenuator over a film, a diode array, or an ionization chamber array^(^ ^4^ ^,^ ^5^ ^,^ ^6^ ^,^ ^7^ ^,^ ^8^ ^)^

The measured curve resembles the depth ionization curve. The energy‐range parameters could be derived from the curve.

In this paper, a method for electron energy constancy test using a wedge attenuator and a computed radiography (CR) system (which fits into the second category as classified above) is described. In this method, the energy constancy (EC) curve, representing the plot of the CR plate signal versus the wedge thickness, is similar to the known depth ionization curve. The energy constancy metric, ${\text{EC}}_{50}$, defined as the thickness of the wedge where the CR plate signal drops to 50% of its maximum, is used as a beam quality indicator. ${\text{EC}}_{50}$ is similar to the $R_{50}$ indicator, which is frequently used for clinical electron beams. We report on the sensitivity and reproducibility of the technique over a period of two years.

## II. MATERIALS AND METHODS

### A. Equipment and measurement geometry

For this study, an acrylic wedge was used in conjunction with a Kodak 2000RT CR system (Kodak Eastman Co, Rochester, NY). The CR system consists of a phosphor storage plate capable of photostimulation, an Agfa fast cassette, and a phosphor plate reader along with its associated software. The plate is used in “indirect detection mode”, and temporarily stores a latent image of the transmitted photon or electron fluence pattern.

The storage phosphors of the CR plate respond to a wide range of irradiation of X‐rays and electrons,^(^ ^9^ ^,^ ^10^ ^)^ and produce a signal that is directly proportional to the energy stored. The CR plate size used in this study is $35\times 43\;{\text{cm}}^{2}$. The pixel size is $0.342\times 0.342\;{\text{mm}}^{2}$ for therapeutic beams; hence, the image has 1024×1240 pixels.

**Figure 1:**
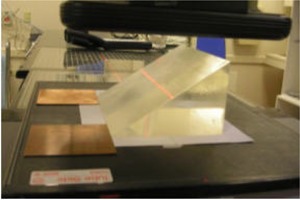
Picture of the measurement setup. The wedge has a height of 10 cm; and a base of $20\times 15\;{\text{cm}}^{2}$, with the 15 cm along the direction of the thickness variation.

The dimensions of the wedge attenuator are shown in Fig. 1. The base of the wedge is 20 cm wide and 15 cm long, its thickness varies from 0 to 10 cm, which is about the practical range for the 20 MeV electron beams. The density of the acrylic wedge is $1.185\;g/{\text{cm}}^{3}$.

Figure 1 shows the measurement geometry. The wedge phantom was placed directly over the CR plate. A $20\times 20\;{\text{cm}}^{2}$ electron cone was used with 110 cm source to phosphorus plate (image) distance (SID). The central axis of the CR cassette was lined up with the crosshairs of the linear accelerator (linac), and with the central axis of the base plane of the wedge. Two copper plates, 1.0 mm thick each, were placed against the thinner edge of the wedge, to facilitate the determination of this edge on the images, as discussed below in more detail. To evaluate the sensitivity of the EC curve to the energy change, polystyrene plates of preset thicknesses were placed on top of the electron applicator to alter the beam quality.

The measurements were performed with the 6, 9, 12, 16 and 20 MeV electron beams available on our Varian Clinac 21EX linac (Varian Medical System, Palo Alto, CA, USA). The beam symmetry and fatness were measured with Profiler and film, and were ±3%, in accordance with our clinical protocol.

### B. Image processing

An IDL (Interactive Digital Language, ITT Visual Information Solutions, Boulder, CO, USA) program was developed to analyze the images and to extract the EC curves.

**Figure 2:**
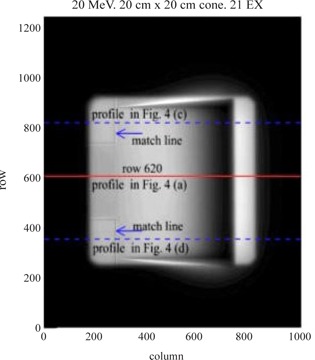
CR image of the wedge irradiated with the 20 MeV beam. The electron field ($20\times 20\;{\text{cm}}^{2}$ cone) edge at right is near column 820, and the thinner edge of the wedge is near column 740. The left electron field edge is near column 200. The solid line corresponds to the cross profile in Fig. 4(a), and the two dashed lines to the cross profiles in Fig. 4(c) and 4(d).

**Figure 3:**
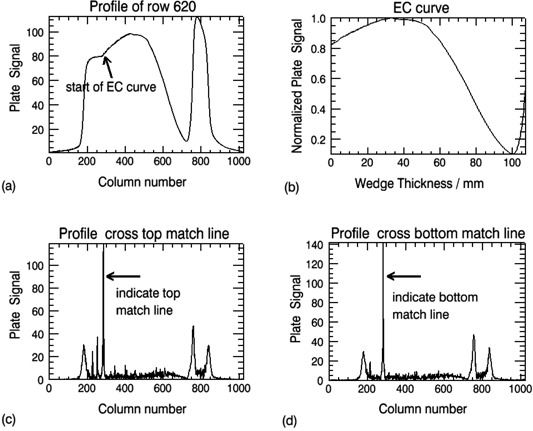
Cross profiles under the wedge phantom for the 20 MeV electrons with $20\times 20\;{\text{cm}}^{2}$ cone: (a) cross profile at the central line of the phosphor plate; (b) EC curve extracted from the profile in (a); (c) and (d) location of columns where the profiles cross the match lines of the copper plates.

An image of the wedge irradiated with the 20 MeV electrons beam using $20\times 20\;{\text{cm}}^{2}$ cone, is shown in Fig. 2. The central line (row 620) is related to the profile in Fig. 3(a). The central axes of the CR cassette were lined up with the central axes of the wedge, so the central row (row 620) of the image was very close to the central line of the wedge phantom. The EC curve extracted from the profile in Fig. 3(a) is shown in Fig. 3(b). From each image, twenty‐one EC curves from profiles centered at row 620 were extracted. Each of the EC curves is part of the profile starting at the wedge's thinner edge. The final EC curve is the average of the 21 curves obtained. Before profile extraction, the CR image was processed using the following schema. To prevent image artifacts due to plate defects or the presence of dust in the CR reader or plate itself, a background image (dark image) was acquired and subtracted from the raw image data. Next, the image was smoothed using a 5×5 pixel filter (Lee filter, an IDL built‐in function^(11)^) to reduce the background noise. The 21 profiles cover an area of about 7 mm wide. The 20 cm width of the wedge is big enough to provide the lateral electron equilibrium, for the highest energy of 20 MeV beam, to the 7 mm wide area.

The starting point of each of the EC curves is the thinner edge of the wedge with zero thickness. To ensure accurate calculation of ${\text{EC}}_{50}$, it is important to determine the position of the thin edge of the wedge precisely (within 1–2 pixels). From the CR image shown in Fig. 2, it can be seen that the thin edge region does not have much contrast. Therefore, two copper plates were placed right next to the thin edge at the two corners. The two match lines can thus easily be detected by the IDL program, which has a built‐in edge enhancement function. The cross lines and the related profiles passing through the match lines, are shown in Fig. 2, and in Fig. 3(c) and 3(d). The cross points at the match lines are indicated by the spikes in Fig. 3(c) and 3(d), respectively. The column number of the starting point of each EC curve is then calculated from the column numbers of the two spikes.

### C. Dose response linearity and processing

The response of the CR plate is linear with dose in the range from 6 to 150 cGy, as shown by Olch.^(12)^ To determine the number of monitor units (MUs) needed for these measurements, EC curves for 6 and 20 MeV beams were obtained with various numbers of MUs. Our data showed that the EC curves were independent of the number of MUs over 20 to 40 MUs for 20 MeV beams, and 30 to 40 MUs for 6 MeV beams. Within the useful data range (i.e. from zero wedge thickness to the thickness where the normalized plate signal drops to 40%) the difference between the 20/30‐MU and the 40‐MU EC curves is generally less than 1%, with a maximum difference of 1.4%. Therefore, 40 MUs were used for all the measurements, which ensure linear dose response and an adequate signal to noise ratio for all energies.

Olch also showed that the standard deviation of the dose response of the CR plate was within 1.5% of the mean in the central area about 7 cm away from the edges. He also found that the loss of the plate signal, when the reading took place within the first minute after irradiation, was negligible. The EC curves required no correction for energy response or readout time delay since the area of interest is more than 7 cm away from the edges and the CR plate was always scanned within 1.5 minutes after irradiation.

The sensitivity of the EC curves versus SID was checked for the 6 MeV beam. The EC curves obtained with 109.5 cm and 110.5 cm SIDs were indistinguishable from the ones with 110 cm SID, showing that the EC curves are not very sensitive to the SID over this range.

### D. Sensitivity and reproducibility

The sensitivity of the EC curve and the related ${\text{EC}}_{50}$ were investigated by inserting different thicknesses of thin polystyrene filters (0.8–2.9 mm) on top of the last scrapper of the electron cone to slightly alter the beam quality.

Reproducibility measurements were carried out on the same Varian 21EX machine. First 12 trials were run to establish the range and the 99% confidence interval of ${\text{EC}}_{50}$ for each of the beam energies. The findings were used as baselines for later measurements during the two‐year period. The same CR plate and the same Agfa cassette were used throughout. Additional experiments were performed using different cassettes and different CR plates to estimate their effects on the determination of the ${\text{EC}}_{50}$.

## III. RESULTS

Figure 4(a) shows that a different cassette, such as a Kodak regular cassette, resulted in quite different EC curves when compared with those obtained using the Agfa cassette, although the same storage phosphor plate was used. When the same cassette was used with different CR plates (same manufacturer), the differences between the curves are minimal, as shown in Fig. 4(b) and 4(c). However, for the 20 MeV beam, plate C resulted in an ${\text{EC}}_{50}$ of 77.65 mm, which is outside the 99% confidence interval. Within the limits of our setup, our experiment showed that no calibration procedures were required as long as the same CR plate and cassette were used.

An example of the EC curves for 6, 9, 12, 16, and 20 MeV electron beams is shown in Fig. 5. These curves are very similar to the depth ionization curves, except at the surface and in the build‐up region where the surface reading of the 6 MeV curve is greater than those of the higher energy curves.

The baseline and the two‐year ranges of ${\text{EC}}_{50}$ are summarized in Table 1, together with median and mean values, standard deviations, and the 99% confidence intervals of the baseline. The two‐year range is either the same as the baseline range or within the 99% confidence interval, which indicates a good reproducibility of the technique. For higher energy beams, the curve near the maximum is fatter and the falloff beyond the maximum is less steep, so the standard deviation increases with the beam energy, though the percentage standard deviations decreases.

The baseline mean values of ${\text{EC}}_{50}$ versus the values of $E_{0}$ (mean incident energy) are plotted in Fig. 6. A strong linear dependence can be inferred. The equation describing the dependence of ${\text{EC}}_{50}$ versus $E_{0}$ is obtained from the least square fitting:

(1) $$EC_{50}(mm)=4.17(mm\cdot MeV^{-1})E_{0}-8.26$$

The measurement system was sensitive to the energy change. The maximum value of the ${\text{EC}}_{50}$ obtained with a preset thickness of polystyrene insert is reported in Table 2. When the thickness was increased to 1.5 mm, the maximum ${\text{EC}}_{50}$ fell outside the 99% confidence interval for 6, 9, and 12 MeV beams; also when increased to 2.3 mm for 16 and 20 MeV beams. This indicates that the beam quality altered by a 1.5 mm polystyrene sheet is detectable for 6, 9, and 12 MeV beams, and when altered by a 2.3 mm sheet for 16 and 20 MeV beams. Assuming the energy loss in polystyrene is 2 MeV/cm, a 2.3 mm sheet would introduce an energy reduction of approximately 0.46 MeV, which can be detected for all the five beam energies. Based on the relationship between mean incident energy and the depth of 50% dose level for electron beams:^(13)^

(2) $${\bar{E}}_{0}(\text{MeV})=2.33(\text{MeV}/cm)\times R_{50}(cm),$$

a 0.46 MeV energy variation can be translated to a 2 mm shift of $R_{50}$, which is the tolerance of the electron beam energy range posed by AAPM TG40.^(1)^

As can be seen in Table 2, the change in ${\text{EC}}_{50}$ versus the change in polystyrene thickness is approximately equal, except for the 20 MeV beam where the change in ${\text{EC}}_{50}$ is only half the change in the thickness. This is probably because the 20 MeV ${\text{EC}}_{50}$ occurs at a wedge thickness of about 7.5 cm, corresponding to a place less than 4 cm from the thicker end of the wedge (the wedge is 15 cm long). The incident electrons near the thicker end retain their energy and may be scattered affecting the ${\text{EC}}_{50}$ of the 20 MeV beam This needs to be further investigated with a larger sized wedge.

**Figure 4(a):**
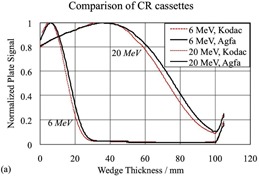
EC curves measured with different cassettes but the same storage phosphor plate for 6 and 20 MeV electron beams; different cassette resulted in different EC curves when all other conditions remained the same.

**Figure 4(b) and (c):**
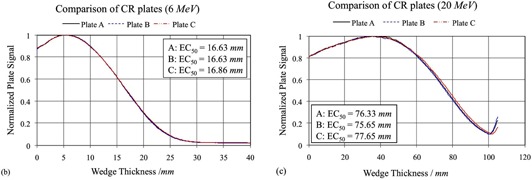
EC curves with different storage phosphor plates (same brand) but the same Agfa cassette.

**Table 1:** Reproducibility of ${\text{EC}}_{50}$.

*Nominal Energy / MeV*	*6*	*9*	*12*	*16*	*20*
Baseline Range / *mm*	16.2−16.9	27.2−28.3	41.2−42.2	57.4−58.8	75.4−76.8
Baseline Mean / *mm*	16.6	27.7	41.5	57.8	75.8
Baseline STDEV / *mm*	0.3	0.3	0.3	0.4	0.4
Baseline STDEV / Mean	1.5%	1.1%	0.7%	0.7%	0.6%
Baseline 99% confidence interval / *mm*	15.7−17.5	26.8−28.6	40.6−42.4	56.6−59.0	74.6−77.0
2‐Year Range / *mm*	16.2−16.9	27.1−28.3	40.8−42.2	57.4−59.0	75.4−77.0

Baseline values and the 99% confidence intervals are based on the first 12 trials. The 2‐year ranges are within the 99% confidence intervals, respectively.

**Figure 5:**
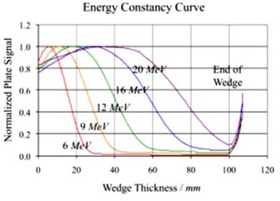
EC curves measured with an acrylic wedge and a CR system, at five beam energies.

**Figure 6:**
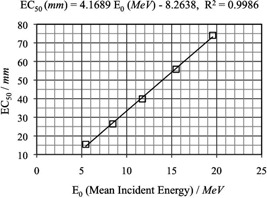
Linear relationship between ${\text{EC}}_{50}$ and $E_{0}$ (mean incident energy).

**Table 2:** Variation of ${\text{EC}}_{50}$ versus the thickness of the polystyrene inserts.

*Thickness of polystyrene inserts / mm*	${\text{EC}}_{50}\;/\;\text{mm}$					
	*6 MeV*	*9 MeV*	*12 MeV*	*16 MeV*	*20 MeV*	*Comments*
0.8	16.2	27.1	41.2	57.4	75.2	Max.
1.5 (+0.7)	15.5(−0.7)	26.5(−0.6)	40.4(−0.8)	56.6(−0.8)	74.8(−0.4)	Max.
2.3 (+1.5)	–	–	39.9(−1.3)	55.8(−1.6)	74.5(−0.7)	Max.
2.9 (+2.1)	–	–	–	–	74.1(−1.1)	Max.
99% confidence interval / *mm*	15.7–17.5	26.8–28.6	40.6–42.4	56.6–59.0	74.6–77.0	Mean±3×STDEV

The ${\text{EC}}_{50}$ values outside the 99% confidence intervals are in bold. Numbers in brackets are the changes from the values in the first row. The changes in the thickness of polystyrene inserts and the ${\text{EC}}_{50}$ values are nearly the same for 6–16 MeV beams.

## IV. DISCUSSION

The combination of a wedge attenuator phantom and the CR system can offer advantages for electron energy check over other methods using a wedge phantom and a detector array because of the imaging characteristics of the CR system. The pixel size of the CR plate is 0.342 mm, while a typical detector array has an inter‐detector separation of 0.4 to 1.5 cm. For the wedge attenuator used in this study, the ${\text{EC}}_{50}$ of the 6 MeV beams is about 16 mm, corresponding to a length of 24 mm on the image. About 70 data points are obtained from this 24 mm region using the CR plate, while only 6 data points would be available for a detector array with a detector resolution of 4 mm. To use a detector array, the wedge attenuator and the system setup must be designed so that a sufficient number of detectors are located within the region of interest. This is especially difficult for low‐energy beams. In addition, the uncertainties due to the relative positioning of phantom and detectors can be large for a coarse sampling grid. For instance, it is rather difficult to determine the maximum value of the depth ionization accurately for low‐energy beams with narrow shoulders.

The width and the length of the wedge phantom used in this study are 20 and 15 cm, respectively. The width is large enough to provide lateral equilibrium for the region of interest for the highest energy beams of 20 MeV. The length is large enough for the measurement of ${\text{EC}}_{50}$ for energies up to 16 MeV. It may need to be increased for 20 MeV beams.

A smaller angle wedge would reduce the filtering effect during the image processing and increase the sensitivity of ${\text{EC}}_{50}$. However, it would occupy a bigger area of the CR plate, and the data points collected would be further away from the central axis. As a result, the uniformity of both the CR plate and the electron field may become a concern.

In this paper we described procedures for system setup, MUs delivery, CR plate scanning, and the image analysis in order to evaluate the beam quality index ${\text{EC}}_{50}$. The steps required to perform a single measurement – set up the CR plate and the wedge in the machine room, irradiation, scanning of the plate – may be perceived as inefficient; nevertheless, the accuracy and reproducibility obtained with the system could overcome that. Obviously, it would be more efficient with a built‐in imaging system, such as EPID, which allows repeated delivering and recording of radiation beams without having to re‐enter the room and perform the scanning at a different location.

## V. CONCLUSIONS

The use of an acrylic wedge combined with a CR system was shown to be a feasible technique to perform monthly electron energy constancy check. A linear relationship was established for the mean incident energy $E_{0}$ and the energy constancy metric ${\text{EC}}_{50}$. The standard deviation of ${\text{EC}}_{50}$ was measured to be from 0.3 to 0.4 mm for different beam energies. The two‐year range of ${\text{EC}}_{50}$ is within the 99% confidence interval established by the baseline data. The ability to detect energy changes of 0.3 MeV for lower energy beams and of less than 0.5 MeV for higher energy beams was demonstrated, which confirmed that this method was able to fulfill the energy constancy check requirements. For those centers equipped with CR system, this method provides a convenient and accurate option for performing monthly QA tasks.
